# The role of cystoprostatectomy in management of locally advanced prostate cancer: a systematic review

**DOI:** 10.1186/s12957-020-1791-5

**Published:** 2020-01-20

**Authors:** Peng Yuan, Shen Wang, Xiao Liu, Xinguang Wang, Zhangqun Ye, Zhiqiang Chen

**Affiliations:** Department of Urology, Tongji Hospital, Tongji Medical School, Huazhong University of Science and Technology, Wuhan, China

**Keywords:** Cystoprostatectomy, Locally advanced prostate cancer, Bladder invasion

## Abstract

**Background:**

The role of cystoprostatectomy for the treatment of locally advanced prostate cancer (LAPC) was evaluated by a comprehensive review of contemporary literatures.

**Methods:**

A systematic search of English language literatures using PubMed, EMBASE, Web of Science, and Cochrane library, from 1990 to 2018, was performed. Two independent authors reviewed abstracts as well as full-text articles and extracted data from the selected manuscripts.

**Results:**

After the literature research, seven articles with a total of 211 patients were identified. Both 120 cases who received cystoprostatectomy for the primary treatment of LAPC and 91 cases for the salvage surgery after local recurrence were finally included. Overall incidence of positive surgical margins ranged from 25 to 78%. The incidence of major complications caused by the surgery during the follow-up time was limited. It had been reported that among LAPC patients who received cystoprostatectomy combined with adjuvant therapies, 5-year cancer-specific survival rate and 5-year biochemical progression-free survival was up to 87.1% and 62.2%. Moreover, symptoms such as hematuria and other urination dysfunctions, as well as patients’ quality of life were significantly improved after cystoprostatectomy in LAPC patients with the bladder invasion.

**Conclusions:**

Cystoprostatectomy can serve as an alternative to the surgical step of multimodal therapy for highly selected LAPC patients with the bladder invasion, which may improve patients’ symptoms and related quality of life. Therefore, cystoprostatectomy as an option for the treatment of LAPC with the bladder invasion may be feasible and safe with considerable survival outcomes.

## Introduction

Prostate cancer is the most common male cancer globally. It poses significant hazards to men’s health, which accounts for 19% of the total estimated new cases of male cancer and ranks the first in USA as per estimate of 2018 [1]. In the meanwhile, the incidence of prostate cancer in China is on the rise especially in better-developed cities [2]. Despite progress in early diagnosis and improving medical interventions, several newly confirmed cases are detected to be locally advanced diseases [3, 4]. It is defined that locally advanced prostate cancer (LAPC) extends beyond the prostate capsule without distant metastasis [5]. And biological recurrence, metastatic progression, and poor survival are associated with LAPC [6–8].

Surgical management is crucial in the multimodal therapy of LAPC combined with adjuvant therapies like radiotherapy and hormone therapy, thus providing patients with individualized treatment basing upon multidisciplinary discussion and cooperation [9, 10]. In recent years, three-dimensional technology and robot-assisted system have enhanced the development of surgical technique and effectively decreased intraoperative and postoperative complications [11, 12]. On the other hand, survival outcomes have also significantly improved as a result of surgery with adjuvant therapies [13–15]. Radical prostatectomy (RP) with extended pelvic lymph node dissection (ePLND) is the most classic surgical treatment of prostate cancer. However, RP can hardly meet both the demands of tumor control and improving function if the bladder neck or urethra is involved [16, 17]. It is worth noting that postoperative complications like urinary incontinence, leakage, and erectile dysfunction can lead to a loss of life quality and even the survival time [18–20].

Cystoprostatectomy and urinary diversion have been recommended as the standard surgical treatment for muscle-invasive bladder cancer. However, when this surgery is applied for LAPC, it may significantly decrease the risk of positive surgical margins in the bladder, improve urinary syndromes, and avoid several urination complications in that the bladder has been removed [21]. It was suggested that surgeons could select cystoprostatectomy with urinary diversion and ePLND as the first step for a multifaceted therapy scheme of LAPC [22]. But on the other hand, some researchers have been worried about there would be excessive treatment in the choice of cystoprostatectomy for prostate cancer patients and possible complications of this surgery [23]. It is equivocal that whether cystoprostatectomy can benefit to patients’ life quality or survival outcomes. Therefore, this paper is to review and summarize current studies on cystoprostatectomy for treating LAPC patients, which aims to further evaluate the clinical significance of the surgery.

## Material and methods

### Inclusion criteria

This systematic review was performed basing on the Preferred Reporting Items for Systematic Reviews and Meta-analyses (PRISMA) guidelines [24]. Studies were selected according to the following criteria:

1. Studies with T3-4N0-1M0 prostate cancer patients who received cystoprostatectomy were included
2. Studies with patients who received cystectomy after RP or along with rectal resection were excluded.

### Search strategy

Two authors (Peng Yuan and Xiao Liu) together performed a computerized comprehensive research on PubMed, EMBASE, Web of Science, and Cochrane library for articles between January 1, 1990 and December 30, 2018. The search terms included locally advanced, T3, T4, prostate cancer, cystoprostatectomy, and cystectomy. It was implemented under a free text protocol. Only English language original articles were included in this study. But the article type of case reports, editorials, letters, review articles, and meeting abstracts were excluded.

### Systematic review and data extraction

After duplicates were removed, two reviewers (Peng Yuan and Xiao Liu) independently screened all abstracts and selected these articles for full-text review. Finally, all necessary data were extracted from full-text articles. If there was any disagreement, a third reviewer (Zhiqiang Chen) was responsible for the final decision. The Newcastle–Ottawa scale [25] was used to evaluate the publications. Articles with scores of 7–9 were defined as high-quality publications, whereas articles with scores of less than 7 were defined as low-quality publications.

### Statistical analysis

The PRISMA flow chart was formulated out by Cochrane RevMan version 5.2 software. Proportions (%) were used for some categorical data.

## Results and discussion

**Fig. 1:**
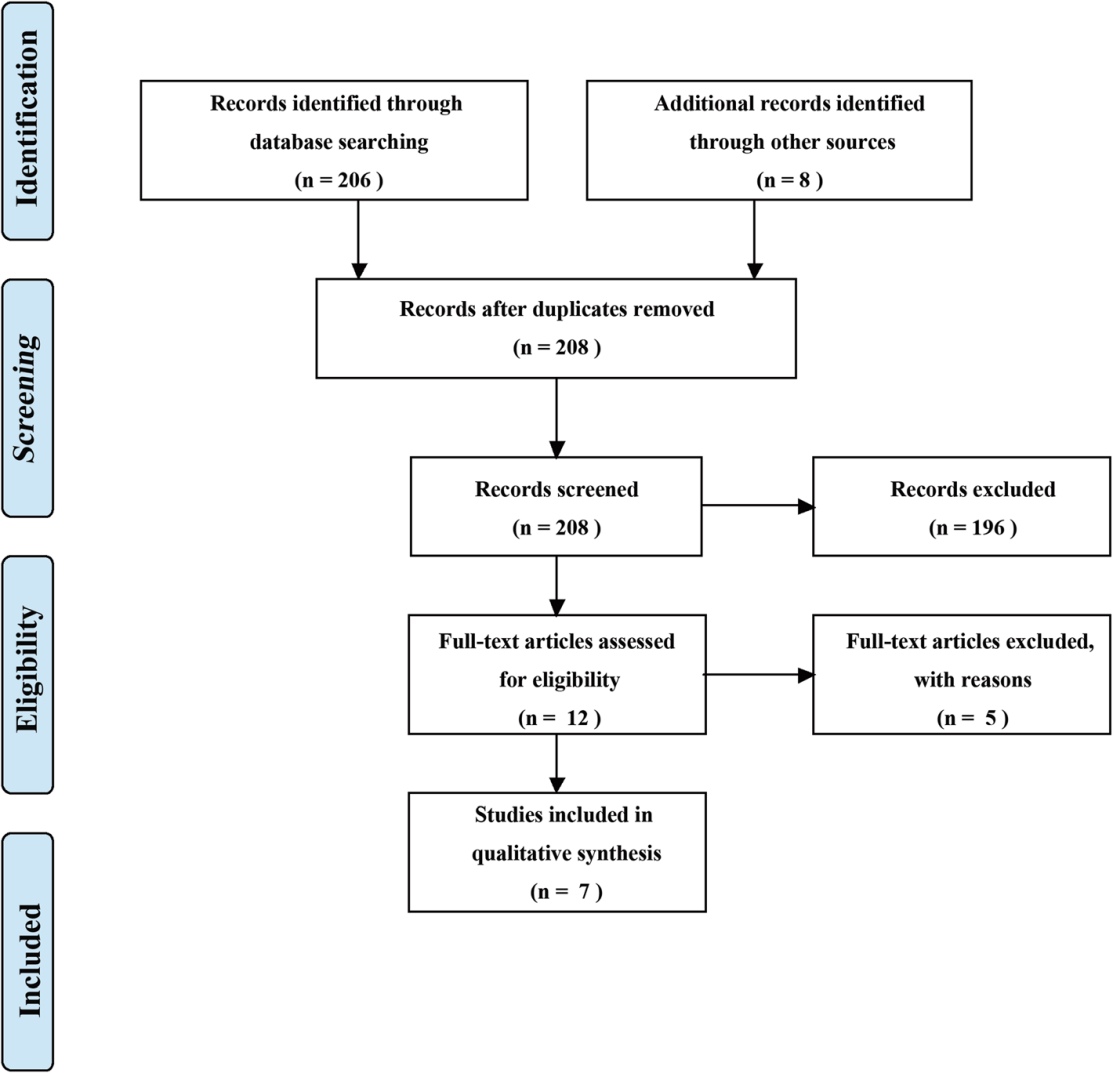
PRISMA flow diagram detailing the search strategy and identification of studies used in data synthesis

A total of 206 articles were identified from the literature research. Seven articles [26–32] were eligible and finally included in this systematic review. The PRISMA flow chart outlining the study selection process was shown in Fig. 1. Seven included articles were all in a retrospective design. According to the Newcastle–Ottawa Scale, six publications scored seven or more and they were regarded as high-quality articles. But one publication [28] was regarded as low-quality article.

### Indication and therapeutic strategy of cystoprostatectomy in LAPC

There were a total of 211 subjects in the final analysis. The baseline data and preoperative results were listed in Table 1. These findings generally confirmed that cystoprostatectomy could be applied to LAPC patients who suffered from the bladder invasion. In addition, it could be chosen as a palliative operation for those who were diagnosed with extensive pelvic prostate cancer metastases or as a salvage surgery for those who had experienced initial radiation failure [33].

**Table 1:** Characteristics and preoperative results of the studies

ID	Study	Time period	Treatment type	*N*	Age (years)	Preoperative treatment	PSA(ng/ml)	Clinical stage
1	Judd et al. 1991	1975–1988	Salvage	4	Nx	RT: 4	Nx	T3N0M0: 3; T4N0M0: 1
			Primary	9	Nx	RT: 1, ADT: 1, ADT: 2	Nx	T3bN_X_M0: 7; T4N_X_M0: 2
2	Edward et al. 1997	1990–1996	Salvage	5	64.2	Nx	12.9	T3aN_X_M0: 2; T3bN_X_M0: 1; T4N_X_M0: 2
3	Sato et al. 2003	1989–2001	Primary	15	64.5	Nx	38.5	T4N_X_M0
4	Leibovici et al. 2005	1995–2003	Salvage	21	68	Chemotherapy: 14	Nx	T4N_X_M0
			Primary	17	60	Chemotherapy: 17	Nx	
5	Ward et al. 2005	1967–2000	Salvage	61	65.3	ADT:43	3.6	T4N_X_M0
6	Kumazawa et al. 2009	1989–2005	Primary	17	64.7	ADT:11	35.3	T4N0M0: 10 T4N1M0: 7
7	Spahn et al. 2017	1972–2011	Primary	62	64.1	ADT:21	35.3	T4N_X_M0

*Nx* not known, *RT* radiotherapy, *ADT* androgen-deprived treatment

Simultaneous ePLND was also required to achieve a better local tumor control [34, 35] and an ideal way of urinary diversion should be comprehensively evaluated based on patients’ conditions as well as personal willing. Patients might undergo expanded resection of rectum and anus if metastatic in very few cases. Kamat et al. found that total pelvic exenteration with urinary and colonic diversion could effectively alleviate the symptoms of perineal pain, hematuria, ureteral obstruction, voiding dysfunction, and rectal incontinence among the patients with locally recurrent prostate cancer and rectal invasion despite initial radiation therapy and hormonal therapy [21].

Open cystoprostatectomy was conducted in all included cases without any application of laparoscopic or robot-assisted surgery. During the operative process, prostate, bladder, urethra, bilateral distant ureter, and bilateral seminal vesicle glands were dissected. Urinary diversion surgeries embraced orthotopic neobladder, ileal conduit, cutaneous ureterostomy, and Kock Pouch. Generally, orthotopic neobladder or ileal conduit was recommended to patients who had fine bowel function without any heavy intestinal disorders, but postoperative complications of intestinal obstruction were possible [26].

Nonetheless, some questions about choosing orthotopic neobladder for LAPC need to be addressed. Among patients with LAPC, adjacent invasions of local tumor have been much complex, which may involve the urethra and other tissues outside the prostate. And there was a high rate of positive margins after cystoprostatectomy in LAPC. More urethra and other adjacent tissues should be intraoperatively removed if possible for maximum tumor clearance. It has been discovered that intraoperative injury of the urethral sphincter and nerve might result in urinary leakage [36, 37]. In addition, postoperative adjuvant radiotherapy can cause severe infection and deteriorate the urination function of the neobladder [38].

So orthotopic neobladder for LAPC patients could be harmful for local recurrence. Moreover, patients were much likely to suffer from bleeding, obstruction, incontinence, and possible infective complications [27]. It was possible that the neobladder was required to be surgically excised if it was terribly invasive or complications were extremely severe without effective control. Taken together, orthotopic neobladder may be discouraged as effective urinary diversion in cystoprostatectomy for the treatment of LAPC. It should be carefully evaluated and selected if the surgery strategy was strongly requested by patients.

Studies found that patients who received cutaneous ureterostomy were at great risk of upper urinary tract obstruction and accompanying urinary infection [28]. According to previous studies, it was also detected that the complications of Koch Pouch were the most reported, and such surgical options had been no longer used in clinical practice. An appropriate urinary diversion method should take into account tumor invasion, patient condition, surgeons’ skill, and patients’ survival expectation [39, 40].

Cystoprostatectomy was implemented in conjunction with therapies such as neoadjuvant hormone therapy, adjuvant hormone therapy, and adjuvant radiotherapy. Adjuvant hormone therapy influenced localized residual tumor, positive lymph nodes and potential metastases, and thus might have improved the survival outcomes [32]. Additionally, patients might receive adjuvant radiotherapy if residual lesions, recurrences, or metastases were detected [30]. Furthermore, it has been reported that cystoprostatectomy with neoadjuvant hormone therapy could conduce to reduce the volume of the prostate and tumor henceforth improving survival outcomes of LAPC patients [31]. On the contrary, it was recommended that neoadjuvant hormone therapy should not be given to LAPC patients who are to receive RP surgery with the consideration of its little improvement on the survival coupled with a significant increase of side effects [41, 42]. Moreover, the surgical complexity in the dissection of the prostate and bladder neck increased because of the possibility of desmoplastic reaction caused by the effect of neoadjuvant hormone therapy. Besides, there was also the well-documented risk of positive surgical margins [43]. Unfortunately, it currently seems unclear whether LAPC patients who intend to receive cystoprostatectomy should attempt neoadjuvant hormone therapy, thus there is need for further clinical study.

### Clinical characterizations and outcomes of cystoprostatectomy in LPAC

No intraoperative death was reported during cystoprostatectomy as the treatment for LAPC patients, while severe intraoperative complications were uncommon in the reported cases. Peri-operative and pathological results of patients were listed in Table 2.

**Table 2:** Perioperative outcomes and pathological results of the studies

ID	Treatment type	Urinary diversion	BLV (ml)	OT (h)	PSM, *n*(%)	PLN, *n*(%)	SVI, *n*(%)	BI, *n*(%)	Gleason score	Overestimated T stage, *n*(%)	Underestimated T stage, *n*(%)
1	Salvage	Ileal conduit: 1 Koch Pouch: 3	5000	9.4	1(25%)	0	1 (25%)	1 (25%)	7.6	0	0
	Primary	Ileal conduit: 8 Koch Pouch: 1	2700	7.8	7(78%)	9(100%)	5 (55.6%)	2 (22.2%)	7.4	4 (44.4%)	0
2	Salvage	Orthotopic neobladder: 2 Ileal conduit: 3	Nx	Nx	3(60%)	2(40%)	5 (100%)	0	8.2	2 (40%)	3 (60%)
3	Primary	Orthotopic neobladder: 8 Ileal conduit: 5 Koch Pouch: 1 Ureterocutaneostomy: 1	Nx	Nx	Nx	Nx	9 (60%)	6 (40%)	Nx	9 (60%)	0
4	Salvage	Orthotopic neobladder: 1 Ileal conduit: 29 Indiana Pouch: 8	2100	Nx	11 (52%)	1 (4.7%)	37(97.4%)	33 (86.8%)	Nx	5 (13.2%)	0
	Primary				5 (29%)	4 (23.5%)					
5	Salvage	Nx	Nx	Nx	20 (33%)	Nx	Nx	Nx	Nx	22 (36%)	0
6	Primary	Orthotopic neobladder: 9 Ileal conduit : 7 Koch Pouch: 1	Nx	Nx	Nx	10 (58.8%)	13 (76.5%)	14 (82.4%)	9.1	3 (17.6%)	Nx
7	Primary	Nx	Nx	Nx	33 (53%)	30 (48.4%)	41 (66.1%)	Nx	Nx	9 (14.5%)	0

*Nx* not known, *BLV* blood loss volume, *OT* operative time, *PSM* positive surgical margins, *PLN* positive lymph nodes, *SVI* seminal vesicle invasion, *BI* bladder invasion

Pathological results were essential in the assessment of the tumor. Gleason scores of tumors in all cases were more than 7. Additionally, 13.2% to 60% of cases in previous studies showed an inconsistency between the pathological stage and clinical stage. Actually, overvaluation was more common than underestimation in the evaluation of the clinical stage. Positive surgical margin was an important indicator of prognosis. Incidences of positive surgical margin in the seven literatures ranged from 25 to 78%.One previous study found that the incidence of positive surgical margins among 62 cases of cT4 prostate cancer patients who had received cystoprostatectomy was 53% [32]. In another study of 114 cases of cT3b-4 prostate cancer patients who had undergone RP, the incidence of positive surgical margin was reported as high as 56.1% [44]. In general, the rate of positive surgical margin of cystoprostatectomy for LAPC is considerable. It is imperative to note that residual tumor of the bladder neck could be avoided in cystoprostatectomy.

The averaged follow-up time in all the seven studies ranged from 21 to 89 months. Postoperative complications and survival outcomes were listed in Table 3. During the follow-up period, postoperative uncommon complications included intestinal injury, wound infection, and systemic infection, but complications of cystoprostatectomy centered around disorders caused by urinary diversion surgery. It was found that complications mainly involved upper urinary tract obstruction, hydronephrosis, and intestinal obstruction. But usually it could well be solved or controlled by drug or surgical management. Nevertheless, complications of bladder neck contraction and urinary incontinence, which were common after RP, could be circumvented in the patients who received cystoprostatectomy [30]. In the context of that, several patients after RP would suffer from severe incontinence for a long time without effective treatment [45, 46]. Otherwise, personal symptoms and discomforts would generally be improved after cystoprostatectomy, especially hematuria and dysuria caused by tumor invasion of the bladder neck. Leibovici et al. demonstrated that cystoprostatectomy could largely alleviate patients’ symptoms and improve the quality of life based on QOL scores [29].

**Table 3:** Postoperative complications and survival outcomes of the studies

ID	Treatment type	Follow-up time (months)	Complications *n*(%)	BR *n*(%)	DM *n*(%)	Survival rate, %	Survival time (months)	Deaths *n*(%)
1	Salvage	32.5	3 (75%)	2 (50%)	1 (25%)	Nx	Nx	0
	Primary	61.8	5 (55.6%)	2 (22.2%)	7 (77.8%)	Nx	Nx	1 (11.1%)
2	Salvage	39.4	Nx	1 (20%)	0	Nx	Nx	0
3	Primary	Nx	1 (6.7%)	Nx	0	5-year CSS: 82% 5-year BPFS : 51%	Nx	0
4	Salvage	21	Nx	Nx	6 (15.8%)	Nx	Median CSS: 31	2 (5.3%)
	Primary	25	Nx					
5	Salvage	49.2	34 (55.7%)	Nx	Nx	10-year CSS: 38%	Average CSS: 52.8	Nx
6	Primary	89	11 (64.7%)	Nx	0	5-year BPFS: 62.2% 5-year CSS: 87.1%	Average OS: 156	9 (52.9%)
7	Primary	35	Nx	Nx	Nx	5-year CSS: 44.5% 5-year OS: 39.8% 7-year CSS: 39.7% 7-year OS: 32.4%	Nx	40 (64.5%)

*Nx* not known, *BR* biochemical recurrence, *DM* distant metastasis, *CSS* cancer-specific survival, *BPFS* biochemical progression-free survival, *OS* overall survival

But as a matter of fact, radical cystectomy is always burdened by a certain percentage of postoperative complications and mortality especially in in the elderly population [47]. Cardiovascular complications, pulmonary embolism, liver failure, sepsis, and severe hemorrhage are blamed for perioperative mortality [48]. Among patients after radical cystectomy for treating bladder cancer, general postoperative complications consist of cardiac, cerebral, pulmonary, vascular and gastrointestinal complications, bleeding, seroma, infection, lymphocele, and renal failure. Meanwhile, there is also a high possibility of urinary diversion related intestinal obstruction, anastomotic stenosis or fistula, ureteric obstruction, hydronephrosis, pyelonephritis, and urinary dysfunction [49–51]. Thereupon, in view of the incidence of complications and potential mortality, surgeons should choose cystoprostatectomy with full consideration for highly selected LAPC patients and explicitly apprise patients of these risks to obtain their informed consent.

Survival outcomes of patients included in this study were listed in Table 3. The survival outcomes after cystoprostatectomy and adjuvant therapies were considerable. Sato et al. discovered that overall cancer-specific survival (CSS) was 82% and biochemical progress-free survival (BPFS) of 5 years was 51% in a series of 15 LAPC patients who received cystoprostatectomy as well as neoadjuvant, adjuvant hormone therapy, or both [28]. What is more, the highest rate of 5-year CSS reached 87.1% in other studies [31].

### Clinical significance of cystoprostatectomy in LAPC

Cystoprostatectomy was of great importance in tumor control, symptoms improvement, and the survival for both the initial treatment of LAPC and salvage surgery after local recurrence. Currently, both surgery and radiation therapy were recommended as a first-line treatment option for LAPC patients [41]. There were always several controversies in treatment tactics with the consideration of patient survival and the quality of life [52–55]. But there was no doubt that surgery could provide accurate pathological stage for patients. Previous studies have demonstrated that there was a difference between pathological stage and preoperative clinical stage of LAPC. The clinical stage was sometimes either overestimated or underestimated [56]. Doctors could not prescribe the best treatment for the patients without accurate diagnosis and tumor stage. Pathological examinations after PLND could offer the exact proof of lymph node status, which determined postoperative treatment and prognosis [57, 58].

Although more and more studies focused on potential benefits of RP as the treatment of LAPC, it was imperative for a realization of postoperative urination complications which profoundly affected personal quality of life [59, 60]. What is more, it was difficult to completely remove the tumor by RP surgery in LAPC with invasion of the bladder. So cystoprostatectomy played a significant role in the tumor clearance and it reduced postoperative urination complications which were caused by the bladder invasion.

### Limitations of cystoprostatectomy in LAPC

RP has been widely used for treating prostate cancer and it is still in constant development. Researchers may question cystoprostatectomy for the therapy of prostate cancer in regards to potential overtreatment. Moreover, cystoprostatectomy was found to have no advantage over RP in the survival outcome [26]. Consequently, these concerns would limit the use of cystoprostatectomy for LAPC. However, it should be well realized that studies on cystoprostatectomy for LAPC were very few. Therefore, it is necessary that more high-quality studies should be designed for the evaluation of cystoprostatectomy in the treatment of LAPC with the invasion of bladder. The differences in complications, survival outcomes, and the quality of life of LAPC patients with the invasion of bladder among cystoprostatectomy, RP, and radiotherapy should be comprehensively observed in further studies.

## Conclusions

In conclusion, cystoprostatectomy, as one of the options for the treatment of highly selected LAPC patients with bladder invasion, can effectively relieve postoperative symptoms and improve the quality of life. Cystoprostatectomy may improve the survival outcomes when combined with adjuvant therapies like hormone therapy or radiotherapy. But more randomized controlled clinical trials with large samples are indispensable to evaluate the value of cystoprostatectomy for LAPC.

## Data Availability

All data generated or analyzed during this study are included in this published article.
